# Conservation genetics of the white‐bellied pangolin in West Africa: A story of lineage admixture, declining demography, and wide sourcing by urban bushmeat markets

**DOI:** 10.1002/ece3.11031

**Published:** 2024-03-01

**Authors:** Koffi Jules Gossé, Sery Gonedelé‐Bi, Sylvain Dufour, Emmanuel Danquah, Philippe Gaubert

**Affiliations:** ^1^ Laboratoire de Biotechnologie, Agriculture et Valorisation des Ressources Biologiques, UFR Biosciences Université Félix Houphouët‐Boigny Abidjan Côte d'Ivoire; ^2^ Centre de Recherche sur la Biodiversité et l'Environnement (CRBE), Université de Toulouse, CNRS, IRD, Toulouse INP Université Toulouse 3 – Paul Sabatier (UT3) Toulouse France; ^3^ SYLVATROP Consulting Conches sur gondoire France; ^4^ Department of Wildlife and Range Management, Faculty of Renewable Natural Resources Kwame Nkrumah University of Science and Technology Kumasi Ghana; ^5^ Centro Interdisciplinar de Investigação Marinha e Ambiental (CIIMAR) Universidade do Porto, Terminal de Cruzeiros do Porto de Leixões Porto Portugal

**Keywords:** conservation genetics, demographic decline, genetic structure, microsatellites, West Africa, white‐bellied pangolin

## Abstract

During the last 40 years, the volumes of African pangolins feeding the illegal wildlife trade have dramatically increased. We conducted a conservation genetics survey of the most traded African species, the white bellied pangolin (WBP; *Phataginus tricuspis*), across three West African countries including Guinea, Côte d'Ivoire, and Ghana. Our study combining mitochondrial DNA sequencing and microsatellite genotyping is the first to reveal a wide pattern of admixture between two of the six mitochondrial lineages as previously delimited within WBP. We found a signature of isolation by distance but a lack of population genetic structuring, supporting the idea that WBP may have underestimated dispersal abilities. Levels of genetic diversity were low in West African lineages (WAfr and Gha) compared to Central Africa, reinforcing the picture of genetic pauperization shared by West African WBP. We observed a 85%–98% decline in the effective population size of WBP occurring c. 3200 to 400 ya, with current numbers (520–590) at the lower end of the conservative thresholds for minimum viable population size. The microsatellite markers were powerful enough to differentiate between individuals and identify replicated samples, confirming the utility of this approach in tracing the pangolin trade. Genetic diversity estimates confirmed that Yopougon, the main bushmeat market from Abidjan (Côte d'Ivoire), was fed by a large trade network as confirmed by vendors reporting 10 different sources situated 62–459 km away from the market. We conclude that WBP distributed in the Upper Guinean Block should be considered a single management unit of high conservation concern, as impacted by genetic diversity erosion, drastic decline in effective population size, and wide range sourcing for feeding urban bushmeat markets. Given the genetic admixture pattern detected within WBP from West Africa, we advocate for a multi‐locus strategy to trace the international trade of the species.

## Graphical abstract

Our work derives from an unprecedented, exhaustive genetic survey of the pangolin trade in three countries from West Africa (Guinea, Cote d'Ivoire, and Ghana). Our study combining mitochondrial DNA sequencing and microsatellite genotyping is the first to reveal a global pattern of admixture between two of the six mitochondrial lineages previously delimited within the white‐bellied pangolin (WBP).

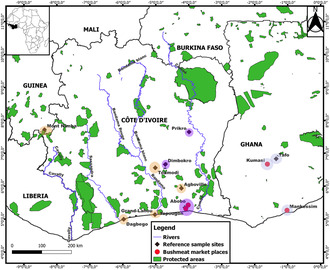

## Résumé

Au cours des 40 dernières années, les volumes de pangolins africains alimentant le commerce illégal d'espèces sauvages ont augmenté de façon spectaculaire. Nous avons mené une étude de génétique de la conservation sur l'espèce africaine la plus braconnée, le pangolin à ventre blanc (PVB; *Phataginus tricuspis*), à travers trois pays d'Afrique de l'Ouest incluant la Guinée, la Côte d'Ivoire et le Ghana. Notre étude combinant le séquençage de l'ADN mitochondrial et le génotypage par microsatellites est la première à révéler un patron généralisé d'admixture entre deux des six lignées mitochondriales précédemment délimitées au sein du PVB. Nous mettons en évidence une signature d'isolement par la distance mais un manque de structuration génétique des populations, soutenant l'idée que le PVB a des capacités de dispersion jusque‐là sous‐estimées. Les niveaux de diversité génétique observés dans les lignées d'Afrique de l'Ouest (WAfr et Gha) sont faibles par rapport à ceux observés en Afrique Centrale, confirmant la paupérisation génétique des populations de PVB en Afrique de l'Ouest. Nous avons observé un déclin de 85 à 98% de la taille efficace de la population ouest‐africaine entre 3200 et 400 ans, les estimations actuelles (520–590) se situant à l'extrémité inférieure des seuils minimums de viabilité. Les marqueurs microsatellites utilisés sont suffisamment puissants pour différencier les individus et identifier les doublons d'échantillons, confirmant l'utilité de ces marqueurs pour tracer le commerce des pangolins. Nos estimations de diversité génétique ont confirmé que Yopougon, le principal marché de viande de brousse d'Abidjan (Côte d'Ivoire), était alimenté par un vaste réseau commercial, comme le confirment les vendeurs signalant 10 sources différentes situées à 62–459 km du marché. Nous concluons que les PVB présents dans le bloc forestier de Haute Guinée devraient être considérés comme une seule unité de gestion à fort intérêt de conservation, étant soumis à une érosion de sa diversité génétique, un déclin drastique de la taille efficace de sa population et un approvisionnement à grande échelle des marchés urbains de viande de brousse. Compte tenu du schéma d'admixture génétique détecté chez les PVB d'Afrique de l'Ouest, nous préconisons une stratégie multi‐locus pour tracer le commerce international de l'espèce.

## 1 INTRODUCTION

Pangolins (Mammalia, Pholidota) are considered the most trafficked mammals in the world (Challender et al., 2015, 2020; Heinrich et al., 2017), with approximately 900,000 individuals seized in the last 20 years (Challender et al., 2020). The eight species of African and Asian pangolins are threatened with extinction, through the cumulative effect of illegal wildlife trade and deforestation (Heighton & Gaubert, 2021). As a consequence, they all are listed in Appendix I of the Convention on International Trade in Endangered Species of Wild Fauna and Flora (CITES; Challender & Waterman, 2017) and rated as Vulnerable, Endangered, or Critically Endangered on the IUCN Red List of Threatened™ Species.

In tropical Africa, land conversion, deforestation, and hunting are the major drivers of faunal depletion, including pangolins (Boakye et al., 2016; Ingram et al., 2018; Pietersen et al., 2014a). Pangolins have long been hunted across their ranges by local communities as bushmeat (Boakye et al., 2014; Zanvo et al., 2021). However, the large demand from the Chinese traditional medicine (Challender & Waterman, 2017; Cheng et al., 2017) and the apparent decrease in numbers of Asian pangolins (Challender & Waterman, 2017; Heinrich et al., 2017) have recently created a global source–sink trade network where pangolin scales (mostly) are being massively exported from Africa to South‐East Asia (Challender & Hywood, 2012; Ingram et al., 2019; Zhang et al., 2020).

From 1972 to 2014, the harvest of African pangolins has dramatically increased. Volumes have been multiplied by nine between 2005 and 2014 (Ingram et al., 2016), while an estimate of >400,000 African pangolins was bound for Asian markets between 2015 and 2019 (Challender et al., 2020). For Central Africa alone, the amount of annually hunted pangolins has been estimated to increase from 0.42 to 2.71 million animals (Ingram et al., 2018). Despite being—wrongly—blamed for their role as intermediate hosts of the COVID‐19 pandemic (Frutos et al., 2020), pangolins were still harvested at high rates (Aditya et al., 2021).

In Côte d'Ivoire (CI), the bushmeat trade remains a vibrant activity despite the national hunting ban established in 1976 (Caspary et al., 2001; Gonedelé Bi et al., 2012). Three species of pangolins can be found on the bushmeat stalls, including the white‐bellied pangolin (*Phataginus tricuspis*), the black‐bellied pangolin (*P. tetradactyla*), and the giant pangolin (*Smutsia gigantea*), although the latter is now subject to local extirpation (Gonedelé Bi et al., 2017). There is growing evidence that part of the pangolin trade in CI is now feeding a non‐domestic, international network, as testified by seizures of scales bound to South‐East Asia and China during the last 15 years (Challender et al., 2020; Challender & Hywood, 2011; Ingram et al., 2019). Recently, Abidjan, the largest city in CI, was highlighted as one of the major western African hubs of the pangolin trade, with a record seizure of >3.5 tons of scales representing c. 10,000–15,000 pangolins (https://www.20minutes.fr/monde/2732399‐20200304‐cote‐ivoire‐saisie‐35‐tonnes‐ecailles‐pangolin‐incineree‐autorites).

Although the white‐bellied pangolin (WBP) is the most trafficked African species (Challender et al., 2020; Mambeya et al., 2018), its natural history remains poorly known, and as a correlate, so remains the genuine impact of the trafficking activities on its populations. Gaubert et al. (2016) described six geographically traceable mitochondrial lineages within WBP, one of which (West Africa; WAfr) being found in Côte d'Ivoire. Such lineage delineation was recently applied to the tracing of the global pangolin trade (Ewart et al., 2021; Zhang et al., 2020). However, fragmentary knowledge on the lineages' precise ranges—notably relative to the neighboring Ghana (Gha) lineage—and the lack of fine‐scale resolution of mitochondrial DNA (mtDNA) markers hinder from addressing the conservation genetics of WBP at a local scale.

Here, we investigate the genetic status of the species across two West African neighbor lineages (WAfr and Gha), building on the assumption that the genetic toolkit is a useful contributor to conservation‐oriented implementations such as population‐based management of pangolins (Zanvo et al., 2022). We set up an approach combining mtDNA to species‐specific, recently developed microsatellites markers (Aguillon et al., 2020), that will contribute to filling the population genetics' knowledge gap that the species remains subject to, notably in West Africa (Heighton & Gaubert, 2021). Our specific objectives were to assess (i) the genetic structure of WBP in West Africa and (ii) their genetic diversity and historical demography. Based on these outputs and an important sampling effort in several major bushmeat selling sites, we eventually discuss the potential of the genetic toolkit to contribute to the tracing of the pangolin trade in the subregion.

## 2 MATERIALS AND METHODS

### 2.1 Sample collection and wet laboratory procedure

**FIGURE 1:**
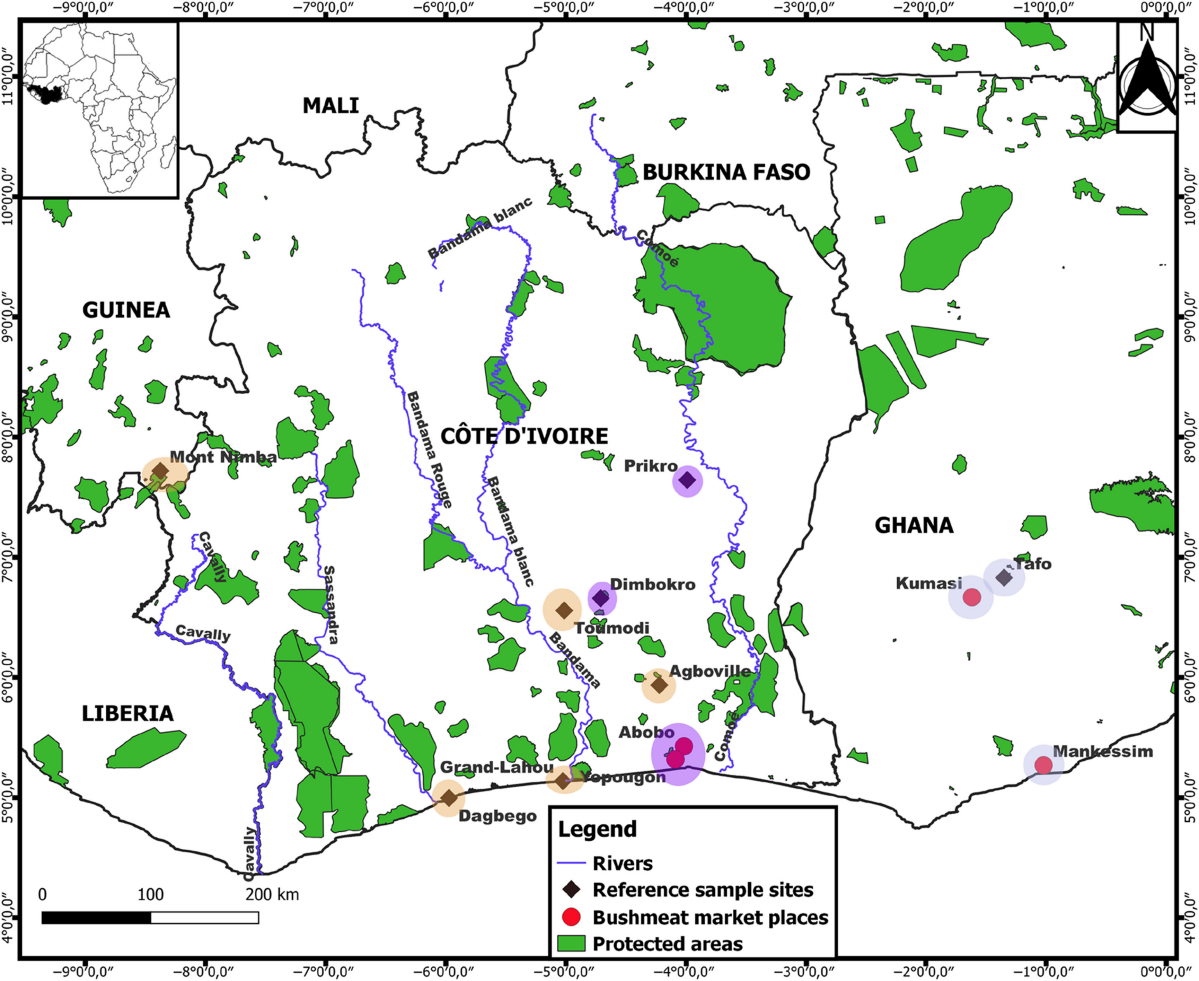
Re‐assessed distribution of the Western Africa and Ghana mitochondrial lineages of white‐bellied pangolins across West Africa. Blue and orange circles correspond to sampling sites where the Ghana (east) and Western Africa (west) mitochondrial lineages occur, respectively. Purple circles mean that both lineages co‐occur. Bushmeat market samples originate from large urban markets and have no precise origin. Reference samples come from circumscribed areas (see Section 2: Material and Methods).

Our study was conducted under research authorization 0632/MINEF/DGFF/FRC‐aska issued by the Direction générale des forêts et de la faune du Ministère des Eaux et Forêts, Côte d'Ivoire. After explaining the objectives of the study, free consent was obtained from market and restaurant vendors prior to sample collection, without providing financial incentives. WBP were identified using the diagnostic characters from body and scales as described in Kingdon (2018). We collected a total of 116 samples from bushmeat markets, restaurants, and food stalls across West Africa, including CI (102 samples; eight localities), Ghana (five samples; three localities), and Guinea (nine samples; one main area; Table S1); so that sampling coverage captures the West Africa (WAfr) and Ghana (Gha) mitochondrial lineages' ranges (Gaubert et al., 2016). The sample scheme was designed so to include both (i) reference samples that will serve for the genetic assessment of WBP (*N* = 33) and (ii) samples from main bushmeat markets (i.e., collected from large urban markets) for assessing trade traceability in the subregion (*n* = 83; Figure 1, Table S1). Reference samples were defined as collected from selling places (rural areas outside of the major urban markets in the city of Abidjan and in Ghana) that sourced pangolins within a <40 km radius (there was no radius overlap among sites). The radius was delimitated through questionnaires addressed to 6–29 sellers from each site. Because our study scale is large and covers c. 1200 km, we consider that such selling places can be used as proxy of “populations,” as long as we could delimitate the proximity of their sourcing localities. However, despite our methodology, we cannot totally exclude the possibility that questionnaires did not fully capture the amplitude of the hunting area feeding these selling places. Samples consisted of fresh or smoked tissues (tongue and muscle), which were preserved in 95% ethanol and stored at 4°C until laboratory processing.

DNA extraction was performed using the NucleoSpin® Tissue kit (Macherey‐Nagel, Hoerdt, France), following manufacturer's recommendations, with 50 μL final elution step repeated twice in order to increase DNA yield. DNA concentrations were estimated with the NanoDrop 1000 spectrophotometer (Thermo Fisher Scientific, Illkirch‐Graffenstaden, France).

We amplified an mtDNA fragment of 432 bp from the control region (CR1), following Gaubert et al. (2016). PCR products were visualized on 1.5% agarose gel and sent for bidirectional sequencing at GenoScreen (https://www.genoscreen.fr/en; Lille, France) and Macrogen Europe (https://dna.macrogen‐europe.com/en; Amsterdam, The Netherlands). Sequences were aligned manually with BioEdit v7.2.5 (Hall, 1999) and deposited in GenBank under accession numbers OP897333–OP897461.

We amplified 20 microsatellite markers developed from the genome of WBP using four PCR multiplexes after Aguillon et al. (2020). Six loci (PT_276641, PT_353755, PT_378852, PT_1162028, PT_1753627, and PT_1849728) from the original multiplexes were not considered in our analysis, because of a significant proportion of amplification failures likely due to fluorescent dye degradation (for each locus, >43% of missing data across all individuals). Serial PCR triplicates were randomly performed to mitigate potential issues related to allele dropout and null alleles in the 14 remaining loci. Consensus was considered achieved when at least two of the three replicates indicated the presence of an allele (Dayon et al., 2020). The PCR products were separated via capillary electrophoresis at GenoScreen.

### 2.2 Genetic analyses—Control region

#### 2.2.1 Phylogenetic clustering

We evaluated the clustering of our CR1 sequences (*N* = 108) from West Africa relative to the six WBP mtDNA lineages (Gaubert et al., 2016) through a distance tree analysis including all the CR1 sequences available in GenBank at the time of drafting the manuscript (*N* = 101). Phylogenetic tree reconstruction was performed in MEGA 11 (Tamura et al., 2021) using neighbor‐joining (NJ) and mid‐point rooting (Farris, 1972), 500 bootstrap replicates, and Kimura 2‐parameter model (Kimura, 1980). In order to assess the impact of the tree rooting strategy on phylogenetic clustering within *P. tricuspis*, we ran a second analysis using *P. tetradactyla* as outgroup with the same parameters.

#### 2.2.2 Genetic diversity and structure

Genetic diversity and structure within WAfr and Gha were reassessed and compared to previous estimates (Zanvo et al., 2022). We used DnaSP v 6.12.03 (Rozas et al., 2017) to calculate for each lineage the number of haplotypes (*h*), mean haplotype diversity (Hd), and mean nucleotide diversity (π). We used network as implemented in POPART v 1.7 (Leigh & Bryant, 2015) to build a haplotype median‐joining network, with *ε* = 0 to minimize the number of alternative median junctions. We subsequently mapped haplotype distribution of the two lineages in QGIS v 3.22.2 (https://changelog.qgis.org/en/qgis/version/3.22/).

### 2.3 Genetic analyses—microsatellites

#### 2.3.1 Genetic diversity

Geneious v 9.0.5 (Kearse et al., 2012) and the Microsatellites plugin (https://www.geneious.com/features/microsatellite‐genotyping/) were used for allele scoring and genotype extraction. After excluding the six deficient loci (see above), we obtained a final dataset of 116 samples genotyped at 14 loci with at most 32% (5 loci) missing data per sample (Table S1).

Quality assessment of our 14 selected microsatellite loci was performed on a subset of “best” 24 samples with ≥80% genotyping success, taken from the whole study zone and which was considered a single “population” (as preliminary results showed a lack of nuclear genetic structure between WAfr and Gha; see below). Detection for the presence of null alleles, assuming population at equilibrium, was performed with Micro‐Checker v 2.2.3 (Peakall & Smouse, 2012). We assessed deviation from Hardy–Weinberg equilibrium for each locus in GenAlEx v 6.503. We performed a permutation test in Arlequin 3.5 (Excoffier & Lischer, 2010) to estimate linkage disequilibrium (LD) between each pair of loci with 1000 randomizations. Bonferroni correction was applied in the three above‐mentioned analyses.

Genetic diversity for each a priori population with *N* ≥ 5 (except in Dagbégo: *N* = 3), including both reference populations (Dagbégo, Dimbokro, Toumodi, and Prikro in Côte d'Ivoire and the area of Mont Nimba in Guinea) and market populations (Yopougon and Abobo in Abidjan, Côte d'Ivoire), was estimated through the number of alleles (Na) and private alleles (pA), observed (Ho), expected (He), and unbiased expected (uHe) heterozygosity (in GenAlEx), allelic richness (A_R_), and Wright's fixation index (*F*_IS_; in FSTAT v 2.9.4; Goudet, 2003). We used the effective number of alleles (Ne; Brown & Weir, 1983), as calculated from the reference populations, as an estimate of genetic diversity to compare among WBP lineages.

#### 2.3.2 Discriminative power of microsatellites markers

We used GenAlEx to evaluate the discriminative power of our microsatellite markers in our total dataset (*N* = 116) by calculating the number of identical genotypes among samples with the *Multi‐locus tagging* option (*Matches* sub‐option). We used the *psex*() function from the *R* package *poppr* (with method = multiple; Kamvar et al., 2014) to assess the probability of encountering the same genotype more than once by chance. We ran Gimlet v1.3.3 (Valière, 2002) to calculate values of unbiased identity probability (uPI) and sibling identity probability (PIsibs), which correspond to the probability that two individuals drawn at random from a population, including (uPI) or not including (PIsibs) siblings, will have the same genotype.

#### 2.3.3 Population structure

We used GenAlEx to perform a principal coordinate analysis (PCoA) with unbiased pairwise genetic distances in order to explore genetic variance among all WBP individuals from West Africa.

We conducted a clustering analysis in STRUCTURE v 2.3.4 (Pritchard et al., 2010) including the 116 genotyped individuals. We performed 20 independent simulations with *K* values ranging from 2 to 8, using 10^5^ Markov chain Monte Carlo (MCMC) iterations and burn‐in =10^4^, assuming admixture model and correlated allele frequencies. We used STRUCTURE HARVESTER v 0.6.94 (http://taylor0.biology.ucla.edu/structureHarvester/) to assess the most likely number of populations (*K*) according to the method of Evanno et al. (2005). The results of the 20 iterations for each *K* value were summarized and averaged with CLUMPAK (http://clumpak.tau.ac.il/contact.html; Kopelman et al., 2015).

Pairwise differentiation between reference populations was estimated by calculating the fixation index (*F*_ST_; Nei, 1986) with a randomization process of 1000 permutations to obtain *p*‐values in Arlequin v 3.5 (Excoffier & Lischer, 2010).

We tested isolation by distance (IBD; Bohonak, 2002) by running a Mantel test with the *adegenet* package in RStudio v 4.0.5 (R Core Team, 2021), where we quantified the correlation (*r*) between genetic (Edward's) and geographic (Euclidean) distances among individuals and populations.

#### 2.3.4 Demographic history

Given the lack of structure observed between WAfr and Gha (see Section 3: Results), we retraced the demographic history of West African WBP as a unique nuclear population using the *R* package *varEff* (Nikolic & Chevalet, 2014), an approximate likelihood method that infers temporal changes in effective population size. We followed Zanvo et al. (2022) in fixing a generation time of 2 years and average mutation rate of 5.10^−4^ per site per Myr. We ran the single mutation, geometric mutation, and two‐phase mutation models, using 10,000 MCMC batches with a length of 1 thinning every 100 lots and JMAX = 3. Proxies of confidence intervals for ancestral and current effective population size estimates were calculated from the harmonic mean distribution of each mutation model.

## 3 RESULTS

### 3.1 Control region

The mid‐point rooted NJ tree based on 209 mtDNA sequences recovered the six WBP geographic lineages (Gaubert et al., 2016) with robust node supports (>80%), including Western Africa (WAfr), Ghana (Gha), Dahomey Gap (DG), Western Central Africa (WCA), Gabon (Gab), and Central Africa (CA; Figure S1a). All the newly produced sequences from Guinea (*N* = 6) and Ghana (*N* = 2) clustered within WAfr and Gha, respectively. The 103 new sequences that we generated from CI clustered both within WAfr (Agboville, Abobo, Dagbégo, Dimbokro, Grand‐Lahou, Prikro, Toumodi, and Yopougon; *N* = 77) and Gha (Abobo, Dimbokro, Prikro, and Yopougon; *N* = 26). The rooted NJ tree (with *P. tetradactyla*) yielded identical clustering patterns (Figure S1b).

**FIGURE 2:**
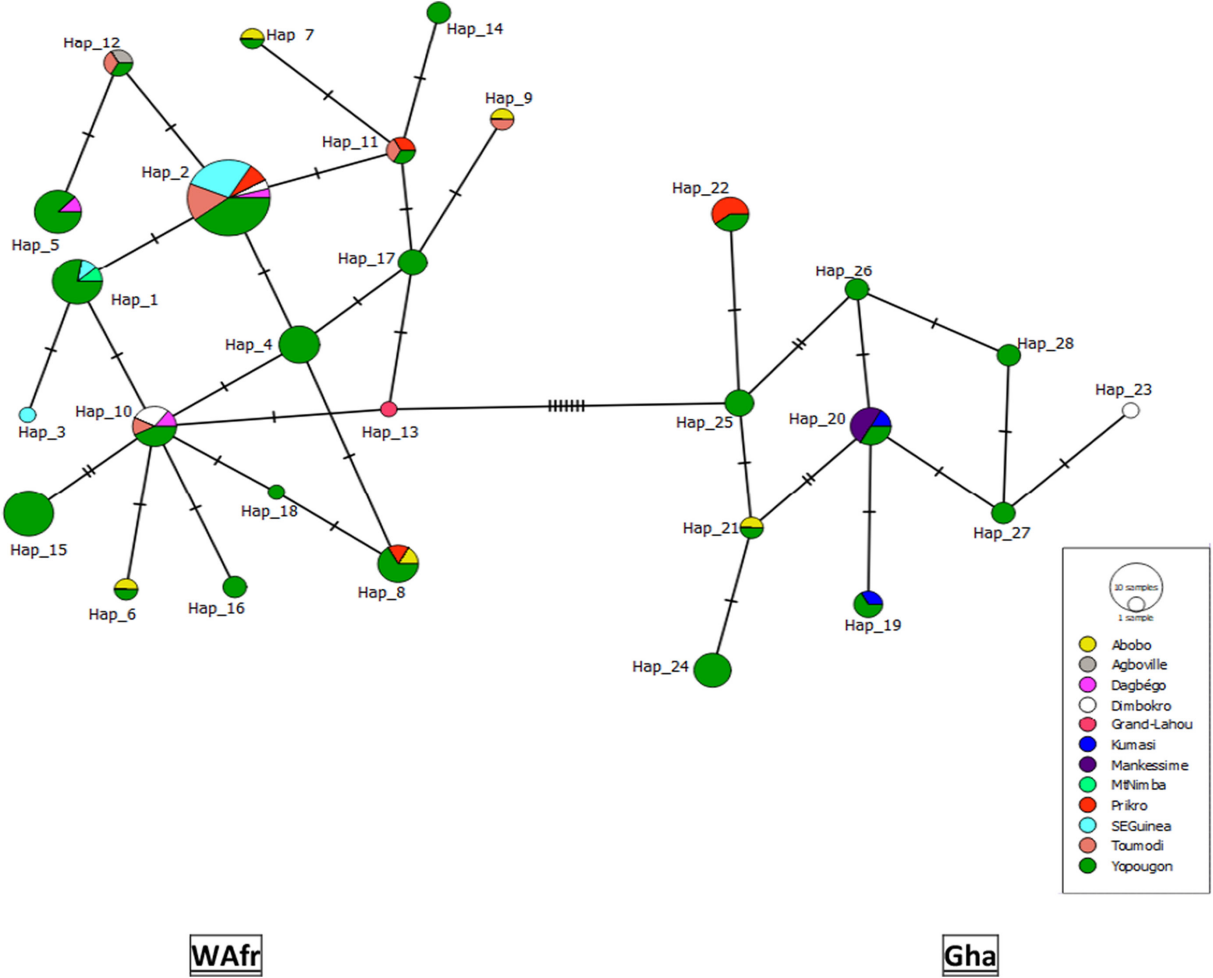
Median‐joining network of control region (CR1) haplotypes across the Western Africa and Ghana mitochondrial lineages of white‐bellied pangolins. Haplotype numbers (e.g., Hap_8) refer to Table S2. Small perpendicular bars represent mutations.

Among WBP mitochondrial lineages, levels of genetic diversity (Pi) were the highest for CA and WCA, and the lowest for West African lineages, WAfr showing the second lowest Pi value after DG (Table 1). In total, 28 CR1 haplotypes were identified across WAfr and Gha (Table S2). The haplotype network showed that WAfr and Gha were separated by 7 mutations, whereas 1–2 mutations separated within‐lineage haplotypes (Figure 2). We did not observe any clear geographic structuring of haplotypes within each lineage. Gha extended into the eastern territory of Côte d'Ivoire (Prikro and Dimbokro; Figure S2). Hap2 was dominant in WAfr (found in 24 samples) and had a large distribution, from SE Guinea to eastern Côte d'Ivoire. Eleven haplotypes were found in the Yopougon bushmeat market, whereas only three haplotypes were unique to the reference populations. Nine Gha haplotypes were found in Yopougon and Abobo markets (Table S2).

**TABLE 1:** Mitochondrial diversity among white‐bellied pangolin lineages.

Lineages	*N*	*S*	*h*	Hd	Pi
				Mean ± SD	Mean ± SD
**Western Africa (WAfr)**	**103**	**15**	**19**	**0.89 ± 0.019**	**0.0054 ± 0.0003**
**Ghana (Gha)**	**41**	**9**	**12**	**0.913 ± 0.018**	**0.0062 ± 0.0003**
Dahomey Gap (DG)	13	8	9	0.936 ± 0.051	0.0051 ± 0.0006
Western Central Africa (WCA)	61	25	32	0.958 ± 0.012	0.0074 ± 0.0005
Central Africa (CA)	11	10	8	0.891 ± 0.092	0.0082 ± 0.0010

*Note*: Lineages re‐assessed as part of this study appear in bold. Gab was not considered as represented by a single sample.

Abbreviations: *h*, number of haplotypes; Hd, haplotype diversity; *N*, number of sequences; Pi, nucleotide diversity; *S*, number of polymorphic sites; SD, standard deviation.

### 3.2 Microsatellites

Five samples shared the same genotype including (i) Yop68, Yop72, and Yop73 and (ii) Yop90 and Yop98. We retained only Yop68 and Yop90 from each respective batch for downstream analysis, as those 100% replicates indicate repeated sampling of two individuals. After removing those replicates, the null hypothesis of encountering the same genotypes more than once by chance was rejected for all pairs of individuals (*p* < .0001).

Two loci significantly deviated (*p* < .003) from Hardy–Weinberg equilibrium (HWE; Table S3), but were not removed from further downstream analyses as deviation from HWE is expected in natural populations and such outlier loci often contribute to the signal of inter‐population structuring (Pearman et al., 2022). No pairs of loci were involved in LD. Null alleles were identified at three loci, involving the two loci deviating from HWE (Table S4). Mean number of alleles (Na) ranged from 3.000 (Dagbégo) to 8.928 (Yopougon), with mean value across populations = 4.673 (Table 2). Observed heterozygosity (Ho) and expected heterozygosity (He) ranged from 0.572 to 0.700 (mean = 0.648) and 0.549 to 0.652 (mean = 0.587), respectively. The values of uHe were similar among populations and market places and ranged from 0.637 to 0.69 (mean = 0.656). *F*_IS_ values were significantly positive for the Dagbego and Mont Nimba populations (0.009 and 0.067, respectively) and Toumodi (0.103). The number of private alleles ranged from 0 to 6 (Mont Nimba) in reference populations and was 1 and 45 in Abobo and Yopougon markets, respectively. Mean effective number of alleles (Ne) across reference populations was 3.099.

**TABLE 2:** Diversity indices estimated from 14 microsatellite markers in reference (bold) and market (bold and italics) sample sites of white‐bellied pangolins from West Africa.

Populations	*Abobo*	Dagbégo	Dimbokro	Mont_Nimba	Prikro	Toumodi	*Yopougon*	All
*N*	5	3	5	9	5	7	73	
Na	3.928	3.000	3.500	4.857	4.214	4.285	8.928	4.673
Pa	1	0	0	6	1	2	45	/
Ho	0.673	0.690	0.660	0.597	0.700	0.572	0.642	0.648
He	0.602	0.549	0.553	0.596	0.583	0.575	0.652	0.587
uHe	0.688	0.690	0.638	0.637	0.637	0.641	0.657	0.656
*F*_IS_	/	0.009	−0.042	0.067	−0.11	0.103	/	/

*Note*: Abobo and Yopougon are urban bushmeat markets.

Abbreviations: *F*_IS_, fixation index; He, expected heterozygosity; Ho, observed heterozygosity; *N*, number of samples; Na, mean number of alleles; Pa, number of private alleles; uHe, unbiased expected heterozygosity.

As calculated from the whole set of genotypes, both unbiased identity probability (uPI) and sibling identity probability (PIsibs) were low (uPI = 7.74e‐15; PIsibs = 1.14e‐05). A number of five microsatellite loci were required to achieve the conservative value of PIsibs <0.01 (Waits et al., 2001; Figure S3).

**FIGURE 3:**
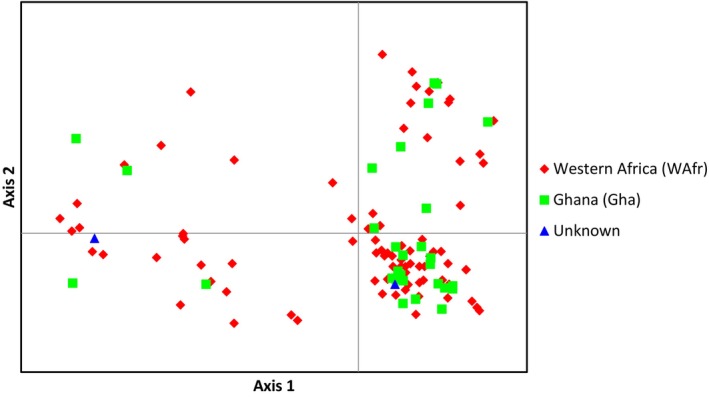
Distribution of nuclear genetic variance (PCoA) within white‐bellied pangolins from West Africa according to mtDNA‐delimited lineages (WAfr and Gha). Axes 1 and 2 explain 11.35% and 8.10% of the total variation, respectively. See Figure S4 for further projections.

The analysis of nuclear genetic variance (PCoA) showed a lack of structuring among mtDNA‐assigned individuals from the WAfr and Gha lineages (Figure 3).

F_ST_ values among reference populations after the Bonferroni correction were significant for the pairs Prikro–Mont Nimba and Toumodi–Mont Nimba. Overall, F_ST_ values among populations ranged from low to moderate levels of differentiation, with a minimum value of 0.00597 (between Dagbégo and Dimbokro) and a maximum value of 0.14879 (between Prikro and Mont Nimba; Table S5).

STRUCTURE identified three optimal clusters (Figure S5). However, from *K* = 2 to *K* = 8 no coherent geographic structuring of populations could be observed (Figure S6).

**FIGURE 4:**
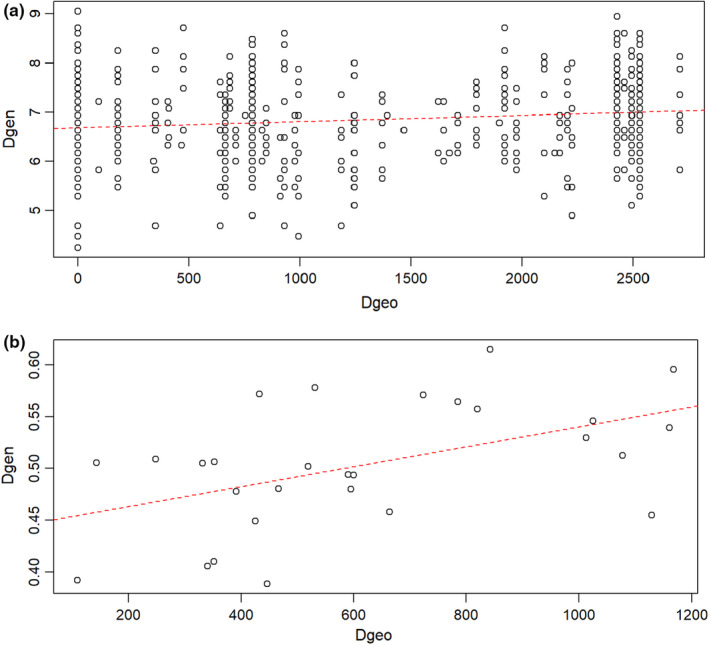
Isolation by distance among (a) individuals and (b) reference populations of white‐bellied pangolins from West Africa, as inferred from 14 microsatellite loci. Dashed curve indicates linear regression. Dgeo: pairwise geographic (Euclidean) distances and Dgen: pairwise genetic (Edward's) distances.

We identified a significant IBD effect among individuals across Western Africa (*r* = .139; *p* = .033) and among reference populations (*r* = .496; *p* = .025; Figure 4).

**FIGURE 5:**
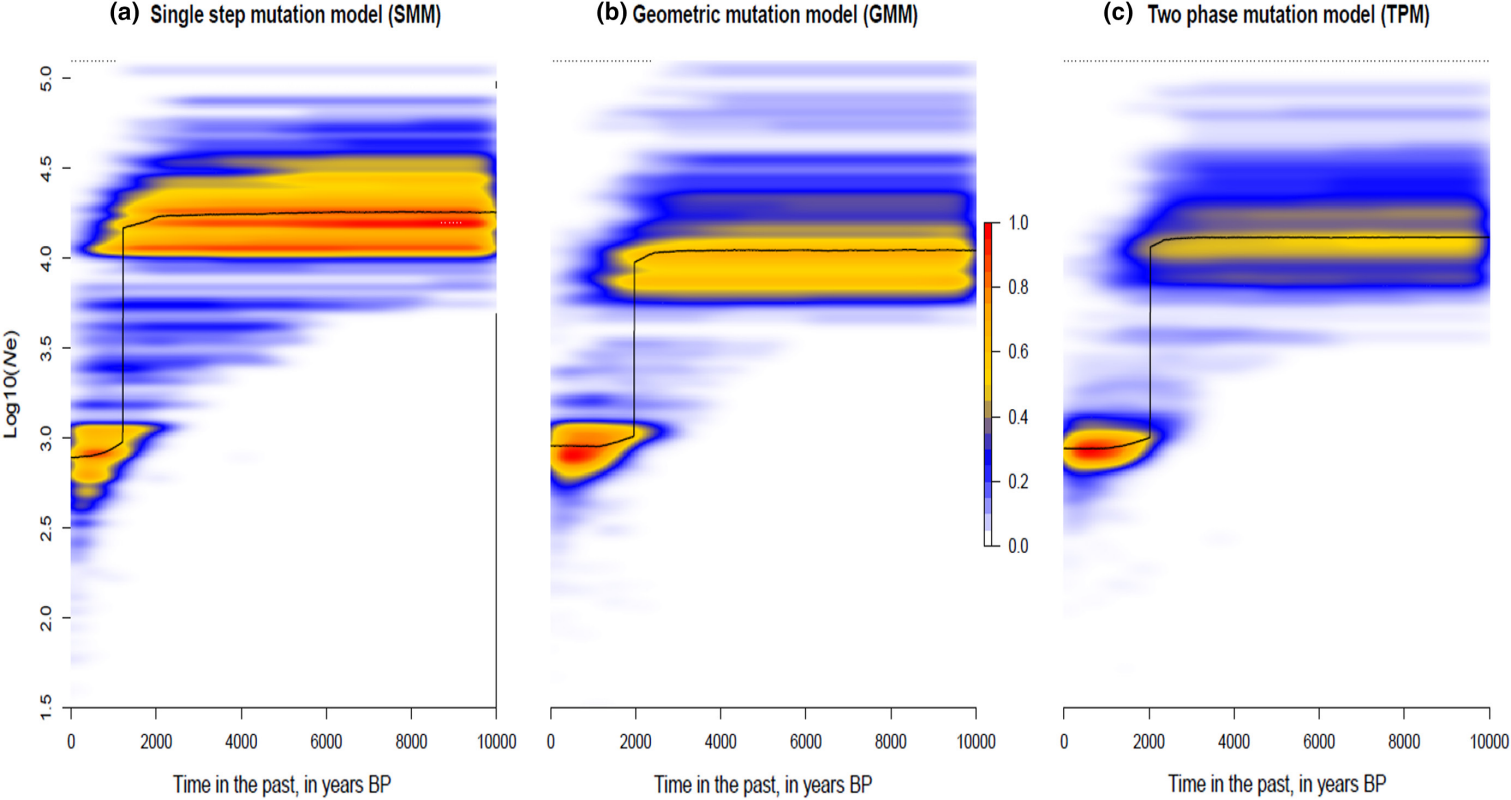
Temporal change in the effective population size of white‐bellied pangolins in West Africa, as estimated from VarEff under three different mutation models. Mode (black line) and kernel density (color scale) of effective population size (*Ne*) posterior distributions are given in years BP.

VarEff showed a drastic decline in the effective population size (*Ne*) of WBP from Western Africa under the three distinct mutation models (Figure 5). Our results suggest 85%–98% reduction of *Ne*, from 10,100–10,900 (ancestral *Ne*) to 520–590 (contemporaneous *Ne*) individuals as harmonic means (95% CI contemponeous *Ne* = 423–5766). Such decline was estimated to occur between 200 and 1600 generations (400–3200 ya).

## 4 DISCUSSION

### 4.1 Lack of genetic structure between West African lineages of white‐bellied pangolins

Our results showed that (i) the WAfr and Gha mtDNA lineages delineated by Gaubert et al. (2016) were geographically overlapping in eastern Côte d'Ivoire and (ii) there was a global pattern of admixture across the West African range of WBP, with no coherent genetic structuring. Our exhaustive sampling across West Africa allows us to re‐delineate the ranges of WAfr and Gha lineages, the latter—previously restricted to Ghana—reaching eastern Côte d'Ivoire. Because we did not detect Gha haplotypes west of the Bandama River and WAfr haplotypes east of the Comoé River, the two rivers may have acted as barriers to the post‐refugial spread of the two mtDNA lineages (see Gaubert et al., 2016). Although previous investigations have suggested that the two rivers could have acted as biogeographic barriers for other terrestrial vertebrates (Leaché et al., 2019; Nicolas et al., 2010), our results are based on a relatively reduced number of samples and should be considered with caution, especially since we obtained a contrasting picture from the microsatellites data (see below).

Beyond revising the delimitation of the two West African mtDNA lineages of WBP, we found a general pattern of nuclear admixture across the West African species' range, where Ghana was not differentiated from the rest of West Africa. This may be due to a high rate of gene flow between the two lineages following range expansion from Pleistocene refugia, the signature of which still being prevalent in the coalescence of mtDNA (see Gaubert et al., 2016). To our knowledge, it is the first time that such scenario is posited as part of the biogeography of West African mammals.

Seminal study on the genetic diversity of WBP in West Africa similarly concluded to a lack of genetic structure within the Dahomey Gap lineage (Zanvo et al., 2022), suggesting unexpected dispersion ability of the species. We detected a significant IBD signature both among individuals and reference populations across West African WBP and a few significant differentiations (*F*_ST_) with the westernmost population from South‐East Guinean forests reinforcing the IBD pattern. However, we should interpret this result with caution, as the Mantel test may not be the most appropriate method to detect IBD, since high *r* values can both indicate intermediate and high dispersal rates (Meirmans, 2015). Moreover, our case study suffers from a lack of baseline information on the dispersal ability of WBP, an uneven sampling of reference populations across the study zone and a possible bias induced by the rural selling places used as “population” proxies, which prevent us from interpreting realistically such IBD pattern. Nevertheless, it is possible that greater than expected dispersion distances are shaping the distribution of genetic diversity of WBP in West Africa (Zanvo et al., 2022). Indeed, long‐range dispersal has been reported and greater than expected mobility was predicted in terrestrial species of pangolins (Ching‐Min Sun et al., 2020; Pietersen et al., 2014b; Van Aarde et al., 1990). As the habitat requirement of WBP notably rely on the presence of large trees (Gaubert, 2011), long‐range dispersal implies continuous forest cover across the study zone, a condition not necessarily met in West Africa due to high rates of deforestation (Aleman et al., 2018). Further studies using tracking devices are necessary to fill the knowledge gaps on the ecology and dispersal ability of the species (Heighton & Gaubert, 2021).

### 4.2 Low genetic diversity and demographic decline in white‐bellied pangolins from West Africa

The level of mitochondrial diversity was low in the Gha and WAfr lineages, coming second after the most genetically depauperate Dahomey Gap lineage. Mean effective number of alleles was slightly above what was found for the Dahomey Gap (3.099 vs. 2.491, respectively; as recalculated from Zanvo et al.'s, 2022 original dataset), whereas it was lower than in Cameroon (3.384; as recalculated from Aguillon et al., 2020's original dataset). Our results show a global pattern of genetic pauperization in WBP from West Africa, compared to Western Central and Central Africa (Aguillon et al., 2020; Gaubert et al., 2016; Zanvo et al., 2022), although nuclear‐based investigations on the species are still preliminary in ranges outside West Africa.

Although levels of inbreeding were in some populations positive and significant, values were low (*F*_IS_ ≤ 0.1) so we cannot conclude that in the case of West African WBP, inbreeding was one of the driving factors for low genetic diversity (contrary to the Dahomey Gap; Zanvo et al., 2022). We posit that admixture between the two previously isolated lineages (WAfr and Gha) could have contributed to counter‐balance, at least partly, the effect of inbreeding in pangolin populations from West Africa (see Keller et al., 2014).

Our analyses concluded to a sharp decline in the effective population size (*Ne*) of WBP from West Africa in the recent past, c. 400 to 3200 ya. We estimated a size reduction of 85%–98%, close to the lower end of the conservative thresholds of minimum viable population size (Clabby, 2010; Reed et al., 2003) but slightly less drastic in amplitude than what was suggested for the Dahomey Gap lineage (Zanvo et al., 2022). The low genetic diversity observed in West Africa may be linked to such recent decline in *Ne* (see Charlesworth, 2009). It remains difficult to relate the period of WBP decline to a specific paleoclimatic or human‐driven event, as both superimpose during the last 3200 years in West Africa and the timing estimate of the event is directly dependent on the mutation rate chosen, which was not specific to pangolins. Since c. 12,000 years, a succession of abrupt periods of drought have affected the West African rainforest zone until 1300–600 ya (Hassan, 1997; Nguetsop et al., 2004). Those, together with the expansion of agriculture c. 2200 ya (Ozainne et al., 2014), could have shaped the decline of WBP in West Africa.

Low genetic diversity and recent, sharp demographic decline can have a deleterious impact on the fitness and survival of West African WBP (see Frankham, 2005; Newman & Pilson, 1997). Additional studies based on nuclear genomic markers (SNPs) will have to be conducted to further improve our estimates of genetic diversity and demographic history of those populations.

### 4.3 Potential of the genetic toolkit to trace the pangolin trade in West Africa

The mtDNA tree showed that all the samples originating from large urban bushmeat markets in Côte d'Ivoire (Abidjan) and Ghana (Kumasi, Mankessim) represented the two lineages (WAfr and Gha) endemic to the study zone. This suggests that in West Africa occur two endemic markets of WBP, one sourcing West African pangolins west of Togo and one sourcing pangolins from the Dahomey Gap, from Togo to south‐western Nigeria (see Zanvo et al., 2022). Such domestic range of the trade contrasts with long‐range trans‐national trade of WBP as observed from seizures (Zhang et al., 2020), notably in Nigeria (Emogor et al., 2021) where multiple WBP lineages amalgamate before exportation (as also detected from seizures in Asia; Ewart et al., 2021). In fact, large bushmeat markets from West Africa might not represent direct hubs for the international trade of pangolins, but rather domestic hubs that source large volumes of pangolins at the national level (and from neighboring countries with close‐by frontiers; Zanvo et al., 2022). However, denser sampling in Ghanaian urban bushmeat markets will have to be conducted to verify this hypothesis, as those markets were represented in our study by only three samples and there is a possibility that WBP from the Dahomey Gap are also sold (from the neighboring Togo).

Genetic diversity indices such as the number of haplotypes and number of private alleles were strikingly high in Yopougon, the main bushmeat market from Abidjan. Such result means that Yopougon, fed by a large sourcing network (Koffie‐Bikpo & Nassa, 2011), is likely selling pangolins from a wide spectrum of locations, in Côte d'Ivoire and possibly Ghana (as Gha haplotypes were also found in the market). This is supported by the 10 different sources cited by vendors when asked about the origin of the pangolins on sale at Yopougon (Table S1), those sources being situated c. 62–459 km away from Abidjan. On the other hand, the high number of private alleles found in Yopougon (45 vs. 0–6 in reference populations) also means that we have not sampled enough reference populations across the study zone to accurately trace the pangolin trade.

Microsatellite markers were powerful enough to differentiate between individuals, and we here confirm that microsatellite genotyping is a theoretically valid approach to apply in pangolin forensics, notably on scale seizures in order to estimate the number of seized carcasses (Singh et al., 2020; Zanvo et al., 2022). We illustrated two cases where samples from a same pangolin were collected twice or thrice in Yopougon. This also proves the utility of our markers to detect upstream bias in sample collection, notably when relying on third parties such a local assistants trained to assist the survey (Din Dipita et al., 2022). It also shows the usefulness of such markers to trace the pangolin trade, where scales and body (meat) often go through different local‐to‐global trade networks (Ingram et al., 2019; Xu et al., 2016; Zanvo et al., 2021). However, the enormous number of scales (reaching several tonnes; e.g., Ewart et al., 2021) found in international seizures may rend unrealistic the applicability of the genotyping approach in counting the number of seized pangolins and trace individual body parts.

The lack of nuclear delimitation between the two West African lineages of WBP has serious implications on the utility of using mtDNA alone as tracer of the pangolin trade. Recently, mtDNA typing following Gaubert et al.'s (2016) lineage delimitations was applied to pangolin scale seizures from Hong Kong (Zhang et al., 2020). The study concluded to the detection of 73 WAfr and 12 Gha samples from two seizures in Kenya and Nigeria. However, from our results any traced individual attributed to WAfr and Gha should be considered as originating from a wider range englobing Ghana as the easternmost possible origin, Côte d'Ivoire and Guinea (and likely the westernmost part of the species' range). Based on the admixture pattern and the lack of genetic structure that we observed, we advocate for a multi‐locus tracing strategy (i.e., involving at least multiple, fast evolving nuclear markers together with a mitochondrial gene) combined with the constitution of extensive DNA registers (reference populations) with denser sampling within populations (see Chakraborty, 1992) and the use of sophisticated tracing approaches modeling the geographic distribution of genetic diversity. More generally, we recommend a multi‐locus approach for tracing the trade of WBP, notably since admixed areas are not uncommon in pangolins (Nash et al., 2018) and can occur elsewhere in the WBP range (Din Dipita et al., 2023).

## 5 CONCLUSION

On the basis of admixture pattern, we suggest that the WAfr and Gha mtDNA lineages could be considered a single management unit (MU; Moritz, 1994; Taylor & Dizon, 1996) of WBP. We showed that this potential MU suffers from genetic diversity erosion and drastic decline in effective population size and is widely sourced by at least one large urban bushmeat market in Côte d'Ivoire. It is also affected by high rates of deforestation (Norris et al., 2010) and a permissive national protection status, notably in Côte d'Ivoire where the species is “partially protected” and can be “hunted and captured by holders of hunting or capture permits within the limits indicated in the permit” (law no. 94–442 of August 16th, 1994 modifying law no. 65–255 of August 4th, 1965 relative to the protection of wildlife and the exercise of hunting). For these reasons, we consider WBP from West Africa as a MU of high conservation concern. Revision of national species status together with law enforcement and awareness campaigns should be urgently conducted, and the conservation status of protected areas reinforced (Gonedelé Bi et al., 2017, 2019).

Our study is the first to provide a comprehensive assessment on the conservation genetics of West African WBP. In practical terms, authorities and stakeholders in charge of sustainable wildlife management from the subregion should take advantage of this level of information to discourage the trade of pangolins. Future research on the natural history and habitat requirement of the species is urgently needed to establish a sounded, cross‐national conservation strategy of WBP in the subregion. Further efforts on the genetics of the species will have to be conducted, both in terms of reference sampling—notably in the westernmost part of the species range (Sierra Leone, Liberia) and in Ghana—and in terms of development of genomic markers (e.g., SNPs) potentially capable of deciphering fine‐scale population structure (Nash et al., 2018), to accurately trace the WBP trade in West Africa.

## AUTHOR CONTRIBUTIONS

**Koffi Jules Gossé:** Data curation (equal); methodology (equal); software (equal); validation (equal); visualization (equal); writing – original draft (equal); writing – review and editing (equal). **Sery Gonedelé‐Bi:** Conceptualization (equal); data curation (equal); funding acquisition (equal); validation (equal); visualization (equal); writing – original draft (equal); writing – review and editing (equal). **Sylvain Dufour:** Data curation (supporting); validation (equal); visualization (supporting); writing – review and editing (supporting). **Emmanuel Danquah:** Data curation (supporting); validation (supporting); visualization (supporting); writing – review and editing (equal). **Philippe Gaubert:** Conceptualization (equal); data curation (equal); funding acquisition (equal); methodology (equal); software (equal); supervision (lead); validation (equal); visualization (equal); writing – original draft (equal); writing – review and editing (equal).

## CONFLICT OF INTEREST STATEMENT

The authors of this work declare that there is no conflict of interest to declare.

## Supporting information


Appendix S1.

## Data Availability

We have uploaded the data as supporting information for reviewers and publication.
