# Supplementary material for: Conservation genetics of the white‐bellied pangolin in West Africa: A story of lineage admixture, declining demography, and wide sourcing by urban bushmeat markets
Source: Ecol Evol. 2024 Mar 1;14(3):e11031. doi: 10.1002/ece3.11031 (PMC10905243; doi:10.1002/ece3.11031)

Appendix Table 1. Meta-data of the samples collected as part of this study, including genotypes based on 14 microsatellites markers. [Excel file]

Appendix Table 2. Haplotype distribution in white-bellied pangolins from West Africa.

In grey, haplotypes belonging to WAfr lineage. In blue, haplotypes belonging to Gha lineage.

| Haplotypes |  |  |  |  | Haplotype count by population / market | | | |  |  |  |  | Sum of haplotype count |
| --- | --- | --- | --- | --- | --- | --- | --- | --- | --- | --- | --- | --- | --- |
|  | Abobo | Agboville | Dagbégo | Dimbokro | Grand Lahou | Kumasi | Tafo | Mankessim | Mont Nimba | Prikro | Toumodi | Yopougon |  |
| Hap 1 | 0 | 0 | 0 | 0 | 0 | 0 | 0 | 0 | 2 | 0 | 0 | 7 | 9 |
| Hap 2 | 0 | 0 | 1 | 1 | 0 | 0 | 0 | 0 | 7 | 2 | 4 | 10 | 23 |
| Hap 3 | 0 | 0 | 0 | 0 | 0 | 0 | 0 | 0 | 1 | 0 | 0 | 0 | 1 |
| Hap 4 | 0 | 0 | 0 | 0 | 0 | 0 | 0 | 0 | 0 | 0 | 0 | 6 | 6 |
| Hap 5 | 0 | 0 | 1 | 0 | 0 | 0 | 0 | 0 | 0 | 0 | 0 | 7 | 8 |
| Hap 6 | 1 | 0 | 0 | 0 | 0 | 0 | 0 | 0 | 0 | 0 | 0 | 1 | 2 |
| Hap 7 | 1 | 0 | 0 | 0 | 0 | 0 | 0 | 0 | 0 | 0 | 0 | 1 | 2 |
| Hap 8 | 1 | 0 | 0 | 0 | 0 | 0 | 0 | 0 | 0 | 1 | 0 | 4 | 6 |
| Hap 9 | 1 | 0 | 0 | 0 | 0 | 0 | 0 | 0 | 0 | 0 | 1 | 0 | 2 |
| Hap 10 | 0 | 0 | 1 | 2 | 0 | 0 | 0 | 0 | 0 | 0 | 1 | 3 | 7 |
| Hap 11 | 0 | 0 | 0 | 0 | 0 | 0 | 0 | 0 | 0 | 1 | 1 | 1 | 3 |
| Hap 12 | 0 | 1 | 0 | 0 | 0 | 0 | 0 | 0 | 0 | 0 | 1 | 1 | 3 |
| Hap 13 | 0 | 0 | 0 | 0 | 1 | 0 | 0 | 0 | 0 | 0 | 0 | 0 | 1 |
| Hap 14 | 0 | 0 | 0 | 0 | 0 | 0 | 0 | 0 | 0 | 0 | 0 | 2 | 2 |
| Hap 15 | 0 | 0 | 0 | 0 | 0 | 0 | 0 | 0 | 0 | 0 | 0 | 9 | 9 |
| Hap 16 | 0 | 0 | 0 | 0 | 0 | 0 | 0 | 0 | 0 | 0 | 0 | 2 | 2 |
| Hap 17 | 0 | 0 | 0 | 0 | 0 | 0 | 0 | 0 | 0 | 0 | 0 | 3 | 3 |
| Hap 18 | 0 | 0 | 0 | 0 | 0 | 0 | 0 | 0 | 0 | 0 | 0 | 1 | 1 |
| Hap 19 | 0 | 0 | 0 | 0 | 0 | 0 | 1 | 0 | 0 | 0 | 0 | 2 | 3 |
| Hap 20 | 0 | 0 | 0 | 0 | 0 | 1 | 0 | 3 | 0 | 0 | 0 | 2 | 6 |
| Hap 21 | 1 | 0 | 0 | 0 | 0 | 0 | 0 | 0 | 0 | 0 | 0 | 1 | 2 |
| Hap 22 | 0 | 0 | 0 | 0 | 0 | 0 | 0 | 0 | 0 | 3 | 0 | 2 | 5 |
| Hap 23 | 0 | 0 | 0 | 1 | 0 | 0 | 0 | 0 | 0 | 0 | 0 | 0 | 1 |
| Hap 24 | 0 | 0 | 0 | 0 | 0 | 0 | 0 | 0 | 0 | 0 | 0 | 5 | 5 |
| Hap 25 | 0 | 0 | 0 | 0 | 0 | 0 | 0 | 0 | 0 | 0 | 0 | 3 | 3 |
| Hap 26 | 0 | 0 | 0 | 0 | 0 | 0 | 0 | 0 | 0 | 0 | 0 | 2 | 2 |
| Hap 27 | 0 | 0 | 0 | 0 | 0 | 0 | 0 | 0 | 0 | 0 | 0 | 2 | 2 |
| Hap 28 | 0 | 0 | 0 | 0 | 0 | 0 | 0 | 0 | 0 | 0 | 0 | 2 | 2 |

Appendix Table 3. Test of deviation from Hardy-Weinberg equilibrium per locus in the Western African lineage of white-bellied pangolins (N = 24).

Significance levels assessed after Bonferroni correction. ns=not significant, (*) P<0.003.

| **Lineages** | **Locus** | **DF** | **ChiSq** | **Prob** | **Signif** |
| --- | --- | --- | --- | --- | --- |
| Western Africa (WA) | PT1453906 | 6 | 56.100 | 0.000 | * |
| Western Africa (WA) | PT739516 | 1 | 24.000 | 0.000 | * |
| Western Africa (WA) | PT34432 | 6 | 17.183 | 0.009 | ns |
| Western Africa (WA) | PT464918 | 15 | 28.995 | 0.016 | ns |
| Western Africa (WA) | PT1973508 | 91 | 118.932 | 0.026 | ns |
| Western Africa (WA) | PT796077 | 78 | 94.820 | 0.095 | ns |
| Western Africa (WA) | PT338821 | 45 | 53.411 | 0.182 | ns |
| Western Africa (WA) | PT619913 | 10 | 13.279 | 0.208 | ns |
| Western Africa (WA) | PT839522 | 55 | 62.000 | 0.241 | ns |
| Western Africa (WA) | PT2019332 | 15 | 18.024 | 0.261 | ns |
| Western Africa (WA) | PT1225378 | 6 | 7.498 | 0.277 | ns |
| Western Africa (WA) | PT308752 | 55 | 60.351 | 0.289 | ns |
| Western Africa (WA) | PT1594892 | 6 | 3.124 | 0.793 | ns |
| Western Africa (WA) | PT1669238 | 21 | 10.099 | 0.978 | ns |

Appendix Table 4. Distribution of null alleles per locus in the Western African lineage (N = 24).

| Locus | Null Present | Oosterhout | Chakraborty | Brookfield 1 | Brookfield 2 |
| --- | --- | --- | --- | --- | --- |
| PT1453906 | Yes | 0.288 | 0.515 | 0.232 | 0.232 |
| PT739516 | Yes | 0.183 | 1 | 0.074 | 0.074 |
| PT619913 | Yes | 0.158 | 0.208 | 0.095 | 0.095 |
| PT796077 | No | 0.071 | 0.074 | 0.064 | 0.064 |
| PT1973508 | No | -0.051 | -0.044 | -0.043 | 0.108 |
| PT839522 | No | -0.071 | -0.064 | -0.064 | 0.166 |
| PT464918 | No | -0.102 | -0.086 | -0.064 | 0 |
| PT34432 | No | -0.533 | -0,2089 | -0.198 | 0 |
| PT1594892 | No | -0.106 | -0.074 | -0.051 | 0 |
| PT308752 | No | -0.019 | -0.017 | -0.016 | 0 |
| PT1669238 | No | -0.075 | -0.065 | -0.060 | 0 |
| PT1225378 | No | -0.003 | 0.006 | 0.004 | 0.210 |
| PT338821 | No | -0.077 | -0.068 | -0.066 | 0.103 |
| PT2019332 | No | 0.001 | 0.014 | 0.011 | 0.251 |

Appendix Table 5. Pairwise F_ST_ values as a measure of differentiation between population pairs across West Africa.

Significance levels assessed after Bonferroni correction (<0.003). ns=not significant, (*) P<0.003

| Population1 | Population2 | F_ST_ | p-value | Signif |
| --- | --- | --- | --- | --- |
| Prikro | Mont-nimba | 0.148 | 0.001 | * |
| Toumodi | Mont-nimba | 0.100 | 0.002 | * |
| Dimbokro | Toumodi | 0.092 | 0.005 | ns |
| Mont-nimba | Dimbokro | 0.075 | 0.005 | ns |
| Prikro | Toumodi | 0.067 | 0.006 | ns |
| Mont-nimba | Dagbégo | 0.077 | 0.006 | ns |
| Toumodi | Dagbégo | 0.035 | 0.010 | ns |
| Dimbokro | Prikro | 0.042 | 0.010 | ns |
| Dagbégo | Prikro | 0.023 | 0.012 | ns |
| Dagbégo | Dimbokro | 0.005 | 0.015 | ns |

Appendix Fig 1a. Neighbor-Joining tree of white-bellied pangolins based on control region (CR1) sequences showing the six mitochondrial lineages clustered after Gaubert et al. (2016). [pdf file]

Bootstrap values >80% are given at nodes. Scale bar represents K2P distance value.

Appendix Fig 1b. Neighbor-Joining rooted tree of white-bellied pangolins based on control region (CR1) sequences showing the six mitochondrial lineages clustered after Gaubert et al. (2016). [pdf file]

Bootstrap values >80% are given at nodes. Scale bar represents K2P distance value.

Appendix Fig 2. Geographic distribution of control region haplotypes across West African white-bellied pangolins.

Size of pie chart is proportional to number of individuals (e.g., 1 individual in Tafo, Ghana).


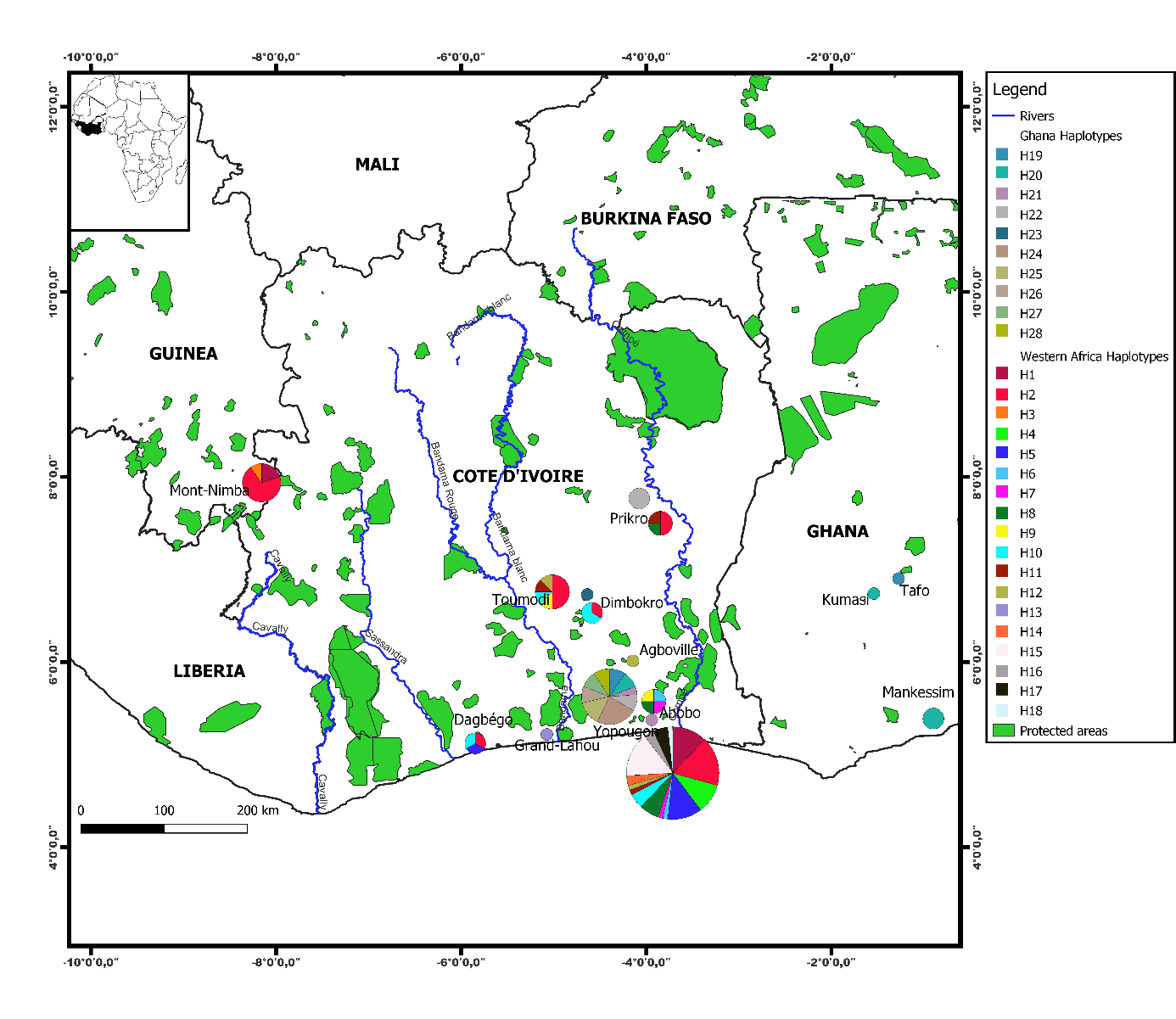


Appendix Fig 3. Unbiased probability of identity (uPI) and probability of identity among siblings (PIsibs) for increasing, optimized locus combinations.

Appendix Fig 4. Distribution of nuclear genetic variance (PCoA) within white-bellied pangolins from West Africa according to mtDNA-delimited lineages (WAfr and Gha) (A and B)

Axes 1, 2 and 3 explain 11.35%, 8.10% and 6.58% of the total variation, respectively.

See Fig 3 for projections on axes 1 and 2.

Appendix Fig 5. Rate of change in the log probability of the microsatellites data between successive K values (Delta K) as calculated and visualized using STRUCTURE HARVESTER.


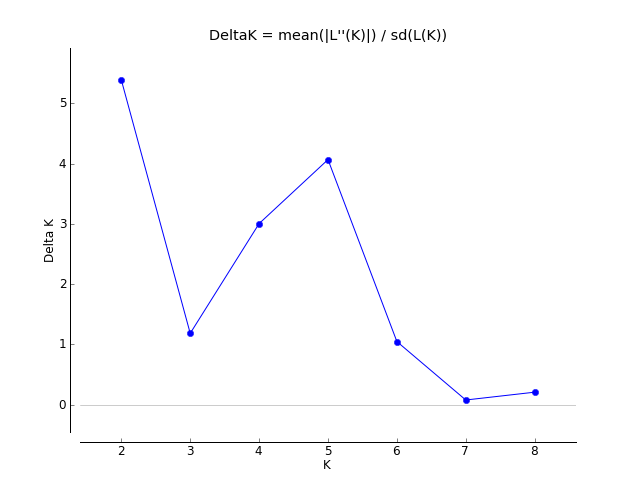


Appendix Fig 6. Plots of the probabilistic individual assignments inferred by STRUCTURE among white bellied pangolins from West Africa.

Vertical axis represents the averaged fraction of ancestry per individual across K=2-8 populations as summarized with CLUMPAK.

Populations as follows: 1- Guinea; 2- Côte d’Ivoire; 3- Ghana.


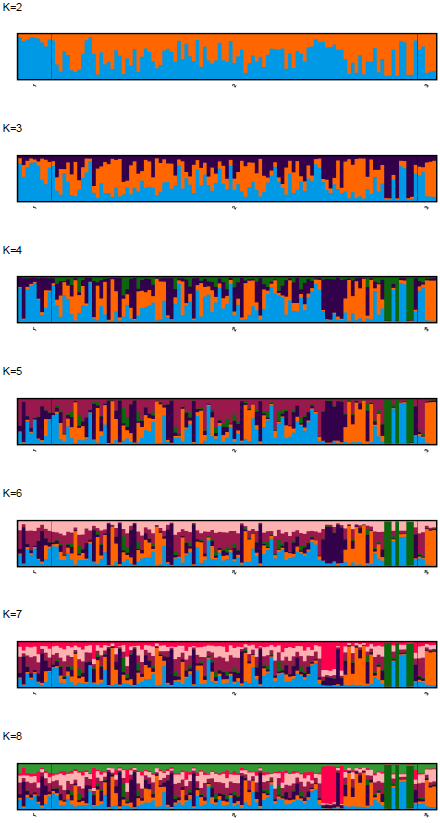

Supplement: Supplementary file 1 — Appendix S1. [file ECE3-14-e11031-s001.zip › ece311031-sup-0001-AppendixS1.docx]
