# Supplementary figures and images for: Conservation genetics of the white‐bellied pangolin in West Africa: A story of lineage admixture, declining demography, and wide sourcing by urban bushmeat markets
Source: Ecol Evol. 2024 Mar 1;14(3):e11031. doi: 10.1002/ece3.11031 (PMC10905243; doi:10.1002/ece3.11031)

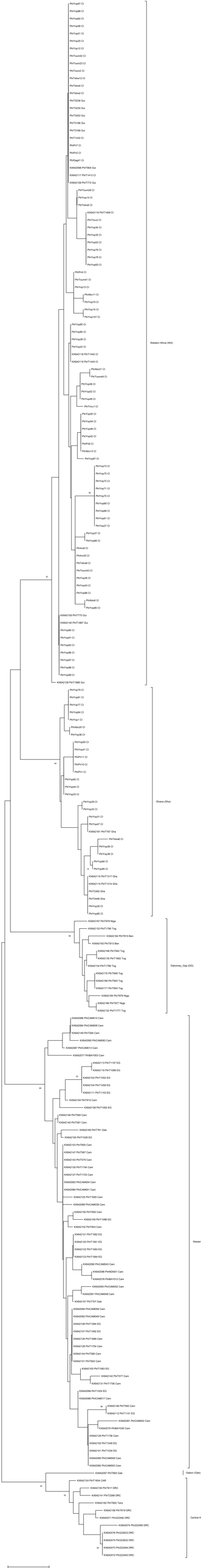

Supplement: Supplementary file 1 — Appendix S1. [file ECE3-14-e11031-s001.zip › ece311031-sup-0002-Appendix_Fig1a.pdf]

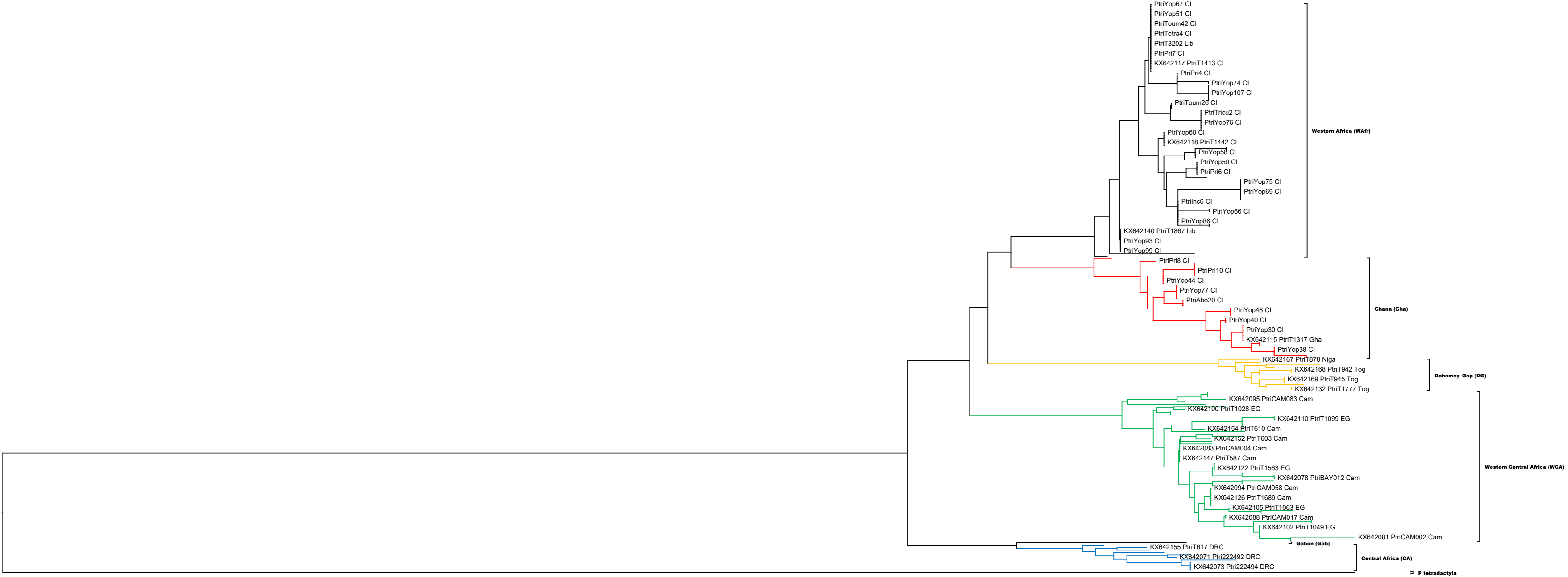

0.01

Supplement: Supplementary file 1 — Appendix S1. [file ECE3-14-e11031-s001.zip › ece311031-sup-0003-Appendix_Fig1b.pdf]
